# Catechin, epicatechin, curcumin, garlic, pomegranate peel and neem extracts of Indian origin showed enhanced anti-inflammatory potential in human primary acute and chronic wound derived fibroblasts by decreasing TGF-β and TNF-α expression

**DOI:** 10.1186/s12906-023-03993-y

**Published:** 2023-06-02

**Authors:** Prakash Monika, M. N. Chandraprabha, K. N. Chidambara Murthy

**Affiliations:** 1 Department of Biotechnology, M.S. Ramaiah Institute of Technology, MSR Nagar, MSRIT Post, Bangalore, 560054 India; 2 Visvesvaraya Technological University, Jnana Sangama, Belgaum, 590018 India; 3 M S Ramaiah Institute of Technology, Center for Bio and Energy Materials Innovation, Bangalore, 560054 India; 4 Neuberg Anand Academy of Laboratory Medicine, Anand Tower, 54, Bowring Hospital Road, Shivajinagar, Bangalore, 560001 India

**Keywords:** Acute wound, Anti-inflammatory activity, Chronic wound, Fibroblasts, Phytocompounds

## Abstract

**Background:**

Although chronic wounds are devastating and can cause burden at multiple levels, chronic wound research is still far behind. Chronic wound treatment is often less efficient due to delay in diagnosis and treatment, non-specific treatment mainly due to lack of knowledge of wound healing or healing resistance genes. It’s known that chronic wounds do not progress towards healing, because it gets stalled in inflammatory phase of wound healing.

**Objective:**

We aimed to use phytoextracts possessing excellent anti-inflammatory properties to regulate the unbalanced levels of cytokines responsible for increased inflammation.

**Methods:**

Evaluation of anti-inflammatory activity of selected phytoextracts namely, *Camellia sinensis* (L.) Kuntze, *Acacia catechu* (L.f) Willd., *Curcuma longa* (L.), *Allium sativum* (L.), *Punica granatum* (L.) and *Azadirachta indica* A. hereafter, called as catechin, epicatechin, curcumin, garlic, pomegranate and neem extracts, respectively in Acute wound fibroblasts (AWFs) and Chronic wound fibroblasts (CWFs) using flow cytometry.

**Results:**

The phytoextracts exhibited no cytotoxicity below 100 μg/ml on normal Human Dermal fibroblasts (HDFs), while garlic extract showed highest cell viability followed by catechin, epicatechin, curcumin, pomegranate peel and neem based on IC_50_ value. Garlic, catechin and epicatechin extracts showed highest anti-inflammatory activities for both TGF-β and TNF-α in both AWFs and CWFs treated cells. After treatment of AWFs with catechin, epicatechin and garlic extracts, TGF-β and TNF-α expression was significantly reduced compared to untreated AWFs and reached to almost normal HDFs level. Also, after treatment of CWFs with catechin, epicatechin and garlic extracts, TGF-β and TNF-α expression was significantly reduced compared to untreated CWFs and was lesser than untreated AWFs.

**Conclusion:**

The present findings reveal the potential of catechin, epicatechin and garlic extracts for the treatment of acute and chronic wounds with excellent anti-inflammatory properties.

## Introduction

Chronic wounds signify a foremost healthcare burden and has an overwhelming impact on illness. They impact on patient quality of life, cost for treatment and remain a major clinical challenge throughout the world [1–3]. Chronic wounds result in greater than 25 billion US$ per year expenditure and is reported in nearly 6.5 million people in US alone [4]. It has been predicted that approximately 1% of the population may develop chronic leg ulcers in the progression of their lifetime [5]. Considering the rise in diabetic patients and other events associated with wound such as accidents, burns and trauma, the global burden is projected to rise [6]. Epidemiology of chronic wounds in India estimated its prevalence at the rate of 4.5/1000 population [7].

Chronic wounds fail to heal within 3 weeks as they do not follow the chronological pattern of wound healing and frequently get ‘stuck’ in inflammatory phase [8]. In order to convert the complex chronic wound into simple healing wound it is important to govern the primary cause of failure to heal in a normal fashion via the regular phases of healing [9, 10]. With the current knowledge of devastating effects of chronic diseases and their mortality rate, there is limited information available on behaviour of different wounds associated with healing resistance. Chronic inflammation, a trademark of the non-healing wound, may eventually render the unhealed wound sites to latent malignant condition [11].

Historically, natural products and substances derived naturally have been used for healing of the wound due to their possession of anti-inflammatory, antibacterial, antioxidant, angiogenic and cell synthesis stimulating potentialities [12, 13]. Phytomedicines may be prepared by combination of two or more herbs, herbal extracts (either alone or in specific combination), and purified plant products (phytochemicals) [14]. Due to their plethora of biological properties, in the current study, we investigate the role of most popular phytoextracts (of Indian origin) known to possess different wound healing properties namely, *Camellia sinensis* (L.) Kuntze [15, 16], *Acacia catechu* (L.f) Willd. [17, 18], *Curcuma longa* (L.) [19–23], *Allium sativum* (L.) [24], *Punica granatum* (L.) [25] and *Azadirachta indica* A. Juss. [26, 27].

By understanding the role of these phytoextracts in cellular level, it is possible to identify the best phytoextract that possess potential anti-inflammatory properties that help to develop therapeutics [13]. For this purpose, two important pro-inflammatory cytokines that are involved in inflammation and wound healing such as TGF-β (Transforming growth factor beta) and TNF-α (Tumour necrosis factor alpha) were considered in the study. In wound healing, TGF-β is vital for inflammation [28] and higher levels of TNF-α has been shown to have a damaging role on healing [29]. Thus, it is very important to have a balance of proinflammatory signals for normal healing of the wound.

## Materials and methods

### Chemicals and cell culture reagents

The commercial phytoextracts used in the study namely catechin, epicatechin, curcumin, garlic, pomegranate, and neem were obtained from Green Chem Pvt. Ltd. Bangalore. HDFs cell line was obtained from American Type Culture Collection, USA. Dulbecco's Modified Eagle Medium (DMEM)—high glucose (#AL111), Foetal Bovine Serum (FBS) (RM10432) and MTT Reagent ((3-(4,5-dimethylthiazol-2-yl)-2,5-diphenyl tetrazolium bromide)) (#4060) were procured from Himedia, TNF-α—FITC Antibody (Cat No: 562082) and TGF-β – PE Antibody (Cat No:562339) were purchased from BD Biosciences, CA, USA. Dimethyl sulfoxide (DMSO) (#PHR1309), Benzyl Penicillin–Streptomycin, Vimentin and Hoechst were purchased from Sigma Aldrich Pvt. Ltd. Alpha Smooth Muscle Actin (α-SMA) was procured from Cell Signalling Technology. Cy3 conjugated secondary antibodies from Abcam, Alexa-488 conjugated secondary antibodies were obtained from Invitrogen.

### Ethical clearance statement, inclusion and exclusion criteria

The conduction of the study was approved by the Institutional Human Ethics committee (MSRMC/EC/AP-07/01–2020). The recruitment of patients was done unbiased and the wound samples were obtained form M S Ramaiah Hospital, Bangalore, India. Acute wounds (less than one week duration) and Chronic wounds (more than 3 weeks duration) were included in the study. Chronic wounds with faecal contamination, gas gangrene and severe bleeding disorders were excluded for the study.

### Isolation and cell culture of primary human AWFs and CWFs

The primary human acute and chronic wound fibroblasts were isolated using the protocol developed by us previously. Here, the explants were allowed to grow undisturbed in a CO_2_ incubator maintained at 37.5 °C, 5% CO_2_ till the fibroblasts attained 80%—90% confluence. The optimized media to grow and culture AWFs and CWFs was DMEM containing 10% FBS, 200 U/ml Penicillin- Streptomycin, 100 μg/ml Gentamycin, 1 μg/ml Amphotericin. The media change for initiation of primary wound associated fibroblast culture is crucial and was done as follows: 50% of the media was removed and fresh media was added (not the entire media change) every alternate day from day 0 of the initiation of the wound fibroblast culture till first trypsinization. Importantly, the explants were not removed from the petridish until the fibroblast monolayer was formed. The cells were observed regularly using inverted bright field microscope (Magnus MLXi Plus, India). The phase contrast images were taken using phase contrast microscope (Olympus 1X71). Fibroblasts were sub cultured once they reached 80%—90% cellular confluence. AWFs and CWFs were grown until 6 passages and freezed in liquid nitrogen at -196 °C for future experiments.

### Immunofluorescence staining

The isolated cells from both acute and chronic wound tissues were characterized based on fibroblast specific markers such as vimentin and α-SMA using Immunofluorescence microscopy. The cells were initially fixed with 3.7% formaldehyde for 15 min at RT and the plates were transferred to 4 °C. The plates were washed with 1X PBS thrice and permeabilized with 0.2% Triton X for 15 min at 4 °C. The blocking buffer containing 20% FSG, 5% Sodium azide, 5% Tween 20 and 1X PBS was added to the plates which was incubated for 1–1.5 h at 4 °C. The blocking buffer was removed, and the plates were dried and labelled using hydrophobic markers to make quadrants. The primary antibodies (1:200 diluted vimentin and α -SMA antibody of mouse origin) were added to each of the respective quadrants and blocking buffer was added to –ve quadrant. The plates with primary antibodies were incubated overnight in cold room. The primary antibodies were removed, and the plates were washed with 1X PBS thrice following which freshly prepared secondary antibodies (1:200 diluted anti-mouse 488 Antibody for vimentin and 1:200 diluted anti-mouse Cy3 Antibody for α -SMA) were added under dark conditions to each of the respective quadrants. The plates were incubated in the dark for 2 h at 4 °C. The plates were finally washed with 1X PBS thrice and stained with Hoechst (1:1000 dilution with PBS made from 1 μg/ml stock) and incubated in dark for 5 min. The Hoechst was removed from the plates, and the plates were further washed with IX PBS and finally mounted. The samples were imaged using an Epifluorescence microscope (Olympus 1X71).

### Cell culture of HDF cell line

HDFs were purchased from the American Type Culture Collection (Rockville, MD, USA). The cells were grown in T25 flask (# 12,556,009, Biolite—Thermo). The cells were maintained in DMEM media containing high glucose and 10% FBS supplemented with 1% antibiotic–antimycotic solution in 5% CO_2_ atmosphere, 18–20% O_2_ at 37 °C in the CO_2_ incubator. Cells were sub-cultured for every 2 days.

### MTT cell viability assay

HDF cell line (standard control) were seeded as 200 μl cell suspension in a 96-well plate at cell density of 20,000 cells per well, without the test phytoextracts. Cells were allowed to grow for about 24 h. 25 μl, 50 μl, 100 μl, 200 μl and 400 μl concentrations of the test phytoextracts were added to the plates and incubated at 37 °C in a 5% CO_2_ atmosphere for 24 h. After incubation, the spent media was removed and MTT reagent of final concentration of 0.5 mg/ml was added and incubated further for 3 h. Following this, MTT reagent was removed and 100 μl of DMSO was added under gentle stirring. The absorbance was measured at 570 nm on a UV spectrophotometer (Shimadzu, Japan). Percentage cell viability was calculated using the formula:

$$\%\;\mathrm{Cell}\;\mathrm{viability}\;=\;\lbrack\mathrm{Mean}\;\mathrm{abs}\;\mathrm{of}\;\mathrm{treated}\;\mathrm{cells}/\mathrm{Mean}\;\mathrm{abs}\;\mathrm{of}\;\mathrm{Untreated}\;\mathrm{cells}\rbrack\;\mathrm x\;100$$

The IC_50_ value was calculated by using linear regression equation i.e., Y = Mx + C. Where, Y = 50, M and C values were obtained from the cell viability graph.

### Anti-inflammatory activity

5 × 10^5^ cells/2 ml cells were seeded in a 6-well tissue culture plate and incubated in a humidified CO_2_ incubator (Healforce, China) at 37 °C for 24 h. After the incubation, the spent medium was removed and treated with 100 μg/ml of experimental phytoextracts (Table 1) and 1 ml of cell culture medium and incubated for 24 h. Untreated cells were maintained as negative control. The cells were harvested and spined down using a table top centrifuge (Remi) for 3 min at 500 × g at 25 °C. The pellet containing cells were washed with PBS and fixed in 1 ml cold 70% ethanol and incubated for 30 min at -20 °C. The cells were further washed with PBS twice following which 5 μl of antibodies were added and the cells were incubated at RT for 30 min under the dark conditions. 500 μl of DPBS mix was added and mixed thoroughly and the cells were acquired by BD FACS Calibur Flow Cytometry and analysed by BD Cell quest pro software. 10,000 cells/events were considered for all the samples and experiments were done in triplicates (*N* = 3). AWFs and CWFs expressing TGF-β and TNF-α before and post treatment with test phytoextracts were measured and the results were compared with normal HDF cell line.

**Table 1:** Details of anti-inflammatory protein marker, test phytoextracts, parameters analysed and concentration used in the study

Sl. No	Name of the commercial phytoextract	Botanical name	Part of the plant used	Major phytoactive compound	Sample code	Cells	Concentration used	Anti-inflammatory protein marker
1	Catechin extract	*Camellia sinensis* (L.) Kuntze	Leaves	Catechin (83%)	CE	AWF & CWF	100 μg/ml	TGF-β & TNF-α
2	Epicatechin extract	*Acacia catechu* (L.f) Willd	Heartwood	Epicatechin (16.6%)	E	AWF & CWF	100 μg/ml	TGF-β & TNF-α
3	Curcumin extract	*Curcuma longa* (L.)	Rhizome	Curcuminoids (95.4%)	CU	AWF & CWF	100 μg/ml	TGF-β & TNF-α
4	Garlic extract	*Allium sativum* (L.)	Bulb	Saponins (NLT 5%)	G	AWF & CWF	100 μg/ml	TGF-β & TNF-α
5	Pomegranate peel extract	*Punica granatum* (L.)	Peel	Polyphenols (NLT 40%)	P	AWF & CWF	100 μg/ml	TGF-β & TNF-α
6	Neem extract	*Azadirachta indica* A. Juss	Leaves	Bitters (NLT 2%)	N	AWF & CWF	100 μg/ml	TGF-β & TNF-α

All the phytoextracts showed absence *of Coliforms, E. coli, Salmonella, Pseudomonas aeruginosa, Staphylococcus aureus*. The presence of toxic heavy metals such as lead, cadmium, arsenic and mercury were NMT 5 ppm. Loss of sample on drying was NMT 5%

### Statistical analysis

Triplicate analysis (*n* = 3) was carried out for each of the experiments, in which data were presented as mean ± SEM. The differences between values analyzed using Statistical significance (P) was calculated by one way ANOVA followed by Dunnett’s test and student paired t-test was executed using GraphPad Prism software. The *P* value ≤ 0.05 was considered as statistically significant.

## Results

### Morphology of AWFs and CWFs

**Fig. 1:**
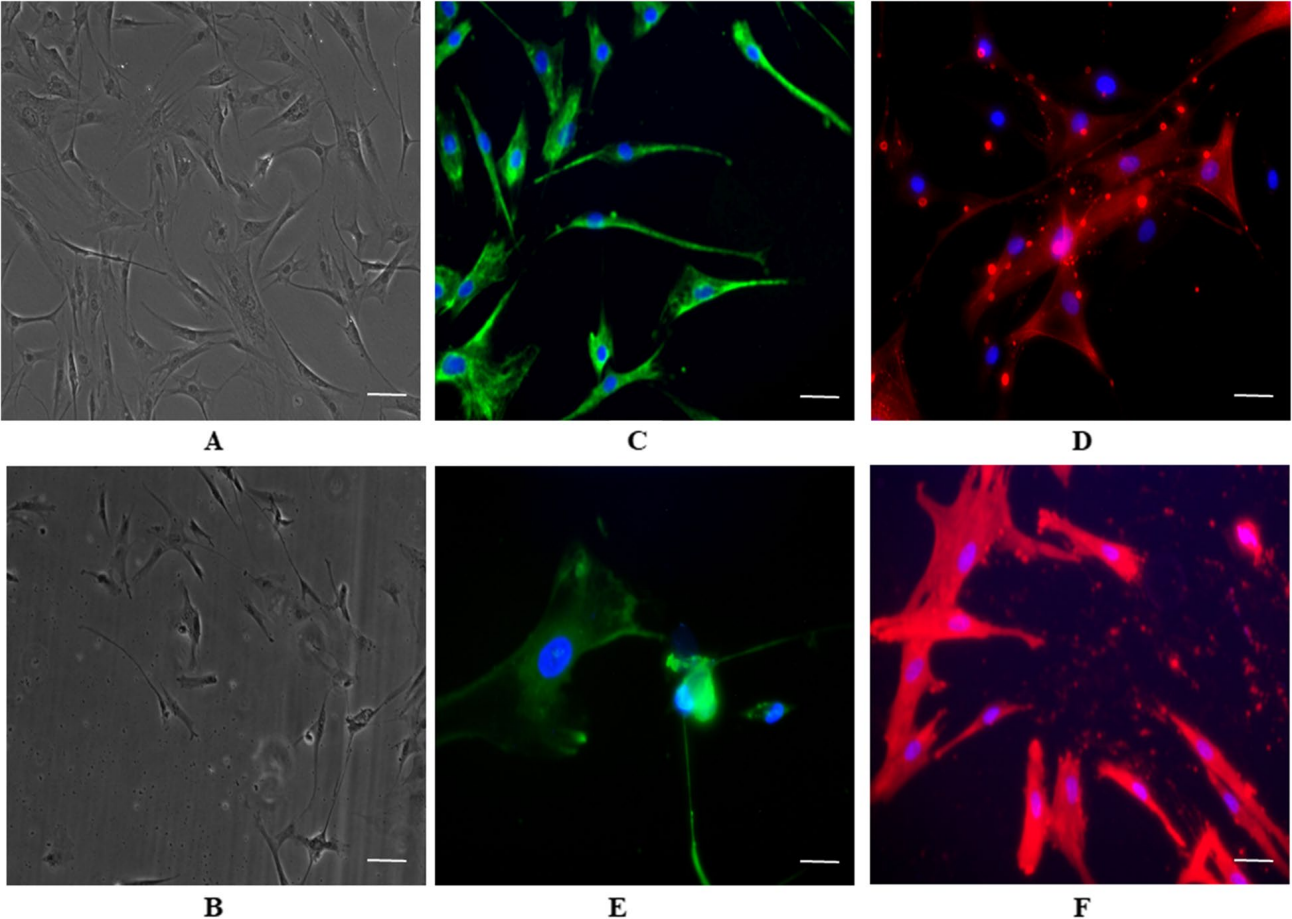
Phase contrast image of AWFs (**A**) and CWFs (**B**) on day 8 of growth of fibroblasts from wound explants, respectively. Images taken at 10 X magnification. Immunofluorescent image of AWFs stained with vimentin (**C**) and α-SMA (**D**). Immunofluorescent image of CWFs stained with vimentin (**E**) and α-SMA (**F**). Images (**C**, **D**, **E**, **F**) are merged and taken at 20 X magnification

The isolated cells from acute and chronic wounds were characterized based on their morphology using phase contrast imaging. The images at 10X magnification showed fibroblasts like cells that had a typical nucleus with 2 or more nucleolus. The cells displayed different morphology such as spindle, fusiform and stellate shape. Few cells had 2 ended spindle shaped elongated structure where as others displayed 3- 10 or more ends that were shorter & narrower. The cells displayed cytoplasmatic prolongations as they interact with neighbouring cells forming cell to cell junction and thus start to proliferate. The cells proliferated and migrated to form monolayer of cells on the plastic surface. The phase contrast microscopic images of AWFs and CWFs are shown in Fig. 1A and B, respectively.

### AWFs and CWFs stained positive for fibroblast specific markers

Vimentin and α-SMA are the frequently employed cell surface protein markers to identify fibroblasts in culture [30]. AWFs and CWFs were stained with fibroblast-specific markers vimentin and α-SMA to determine the identity of the isolated cells from wounds as particularly fibroblast cells. The isolated cells (both AWFs and CWFs) were found to be pure population of fibroblasts, as observed by the expression of fibroblast-specific markers in immunocytochemical analysis experiments. The immunofluorescent images of AWFs and CWFs stained with vimentin and α-SMA are shown in Fig. 1C, D and E, F, respectively.

### Cell viability of test phytoextracts treated AWFs and CWFs

**Fig. 2:**
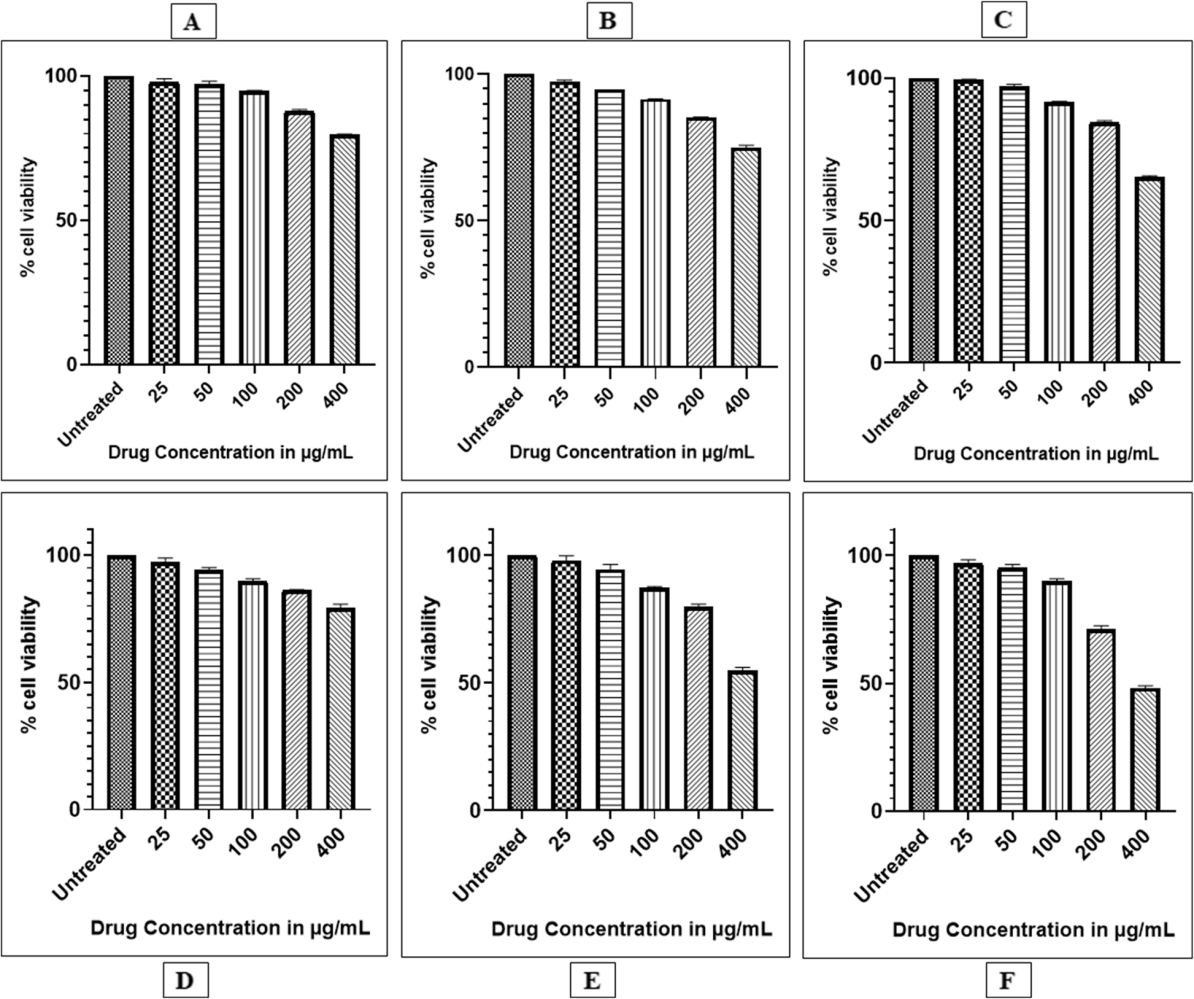
Effect of Catechin (**A**), Epicatechin (**B**), Curcumin (**C**), Garlic (**D**), Pomegranate (**E**), Neem extracts (**F**) on cell viability of HDF cell line

**Fig. 3:**
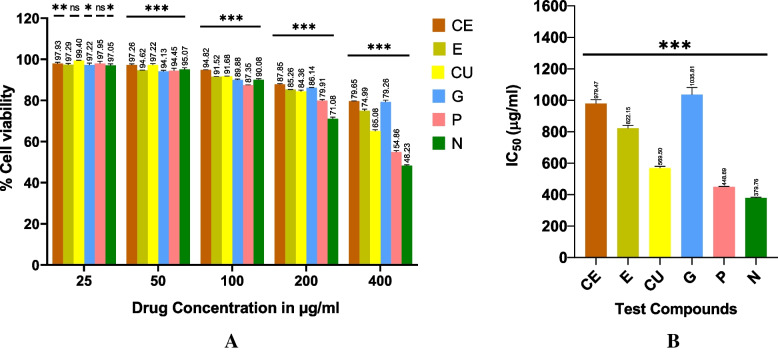
Effect of test phytoextracts on cell viability of HDF cell line (**A**) IC_50_ value of phytoextracts against HDF cell line (**B**). Note: Statistical significance (P) calculated by one-way ANOVA followed by Dunnett’s test—*P* < 0.05 was considered as statistically significant

MTT assay was carried out to probe the effect of phytoextracts such as catechin, epicatechin, curcumin, garlic, pomegranate, and neem on cell viability of normal HDFs. Various concentrations (25, 50, 100, 200 and 400 μg/ml) were used to evaluate the toxicity of the test phytoextracts on normal HDFs. IC_50_ represents the concentration at which a substance exerts half of its maximal inhibitory effect. IC_50_ value of the phytoextracts was calculated using GraphPad Prism software version 8.0.1. Percentage cell viability of HDFs treated with various concentrations of test phytoextracts is given in Table 2. Reduced cell viability was observed in a dose dependent manner after treatment with phytoextracts (Fig. 2) and the effect of phytoextracts on cell viability of HDFs is shown in Fig. 3A. Phytoextracts were considered to be safe if they had cell viability value > 85%. The IC_50_ values of catechin, epicatechin, curcumin, garlic, pomegranate and neem extracts were 979.5 ± 24.39, 822.2 ± 18.55, 569.5 ± 10.89, 1036 ± 45.67, 448.7 ± 2.95 and 379.8 ± 4.10 μg/ml, respectively against HDFs (Table 3). The IC_50_ values of test phytoextracts against HDFs is shown in Fig. 3B.

**Table 2:** Percentage cell viability of HDF cell line treated with various concentrations of phytoextracts

Test phytoextracts	Cell viability (%)					
	**Untreated** **(control)**	**Concentration of test phytoextracts**				
		**25 μg/ml**	**50 μg/ml**	**100 μg/ml**	**200 μg/ml**	**400 μg/ml**
**CE**	100 ± 00	97.93 ± 0.62 **	97.26 ± 0.47 ***	94.82 ± 0.09 ***	87.85 ± 0.27 ***	79.65 ± 0.08 ***
**E**	100 ± 00	97.29 ± 0.69 **	94.62 ± 0.09 ***	91.52 ± 0.10 ***	85.26 ± 0.09 ***	74.99 ± 0.79 ***
**CU**	100 ± 00	99.4 ± 0.14	97.22 ± 0.63 **	91.68 ± 0.12 ***	84.36 ± 0.68 ***	65.08 ± 0.66 ***
**G**	100 ± 00	97.22 ± 0.93*	94.13 ± 0.54 ***	89.88 ± 0.51 ***	86.14 ± 0.14 ***	79.26 ± 0.86 ***
**P**	100 ± 00	97.95 ± 1.09	94.45 ± 1.13 ***	87.35 ± 0.27 ***	79.91 ± 0.53 ***	54.86 ± 0.73 ***
**N**	100 ± 00	97.05 ± 0.73*	95.07 ± 0.81 ***	90.08 ± 0.45 ***	71.08 ± 0.83 ***	48.23 ± 0.49 ***

Values are expressed as the mean ± SEM (*n* = 3); Statistical significance (P) calculated by one-way ANOVA followed by Dunnett’s test— ***P < 0.001, **P < 0.01, *P < 0.05 were considered as statistically significant by comparing treated group with control group

**Table 3:** IC_50_ value of phytoextracts against HDF cell line

Test phytoextracts	IC_50_ (μg/ml)
CE	979.5 ± 24.39***
E	822.2 ± 18.55***
CU	569.5 ± 10.89***
G	1036 ± 45.67***
P	448.7 ± 2.95***
N	379.8 ± 4.10***

Values are expressed as the mean ± SEM (*n* = 3). Statistical significance (P) calculated by one-way ANOVA followed by Dunnett’s test— ***P < 0.001 was considered as statistically significant

Garlic extract had highest IC_50_ value, followed by catechin, epicatechin, curcumin pomegranate peel and neem (Fig. 3B). From the findings, it is apparent that garlic extract showed least toxicity and neem extract showed highest toxicity to normal HDFs at the treated highest concentration of 400 μg/ml. These findings suggest that garlic extract was found to be the safest among all other phytoextracts. However, all the test phytoextracts were considered to be safe for use since the IC_50_ value of these were above 300 μg/ml. All test phytoextracts considered in the current study exhibited no cytotoxic effect below 100 μg/ml and thus, the non-cytotoxic concentration of 100 μg/ml was selected for both AWFs and CWFs for further mechanistic studies.

### Anti-inflammatory activity of selected phytoextracts

**Fig. 4:**
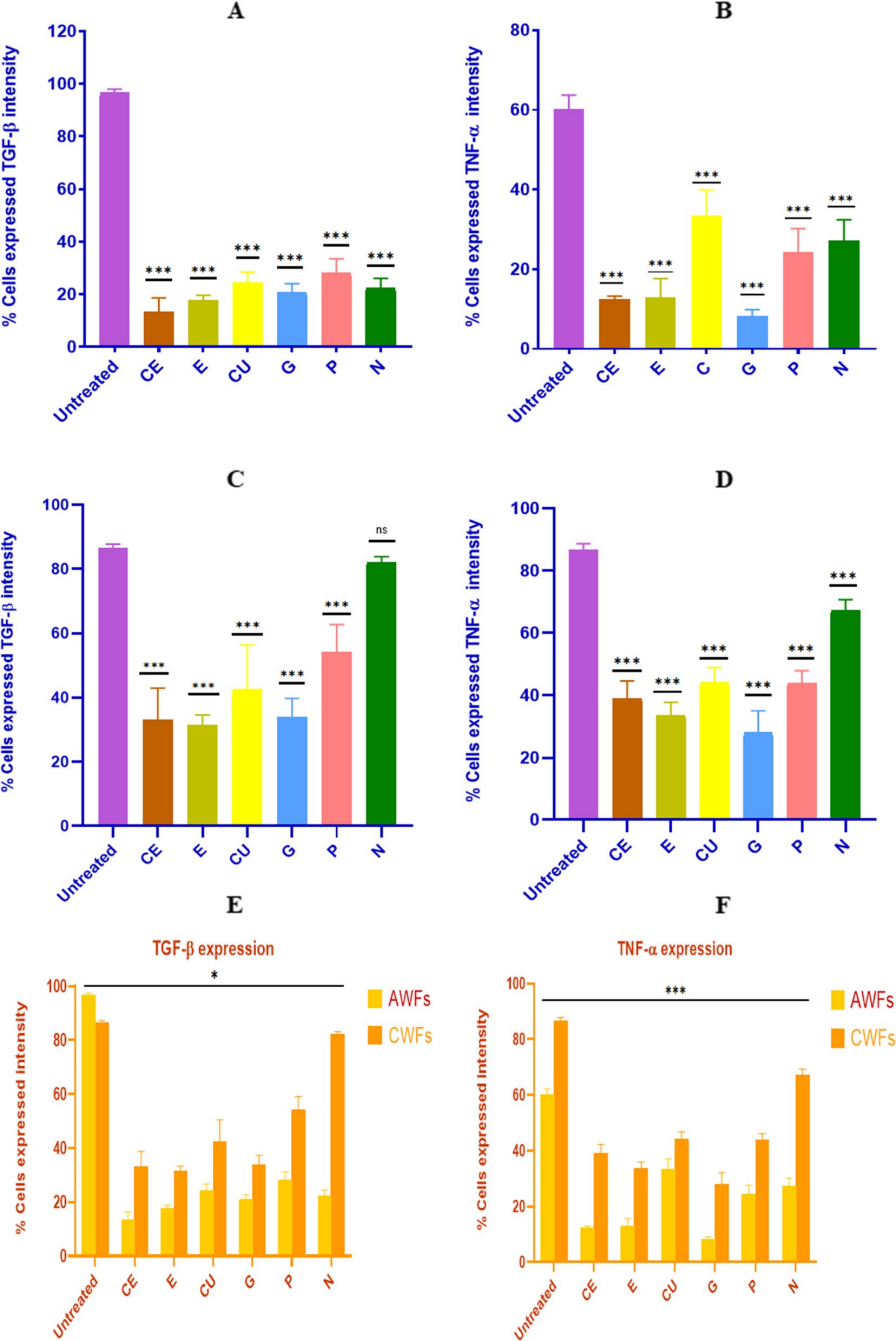
TGF-β expression against AWFs (**A**), TNF-α expression against AWFs (**B**), TGF-β expression against CWFs (**C**), TNF-α expression against CWFs (**D**) Comparative expression of TGF-β (**E**) and TNF-α (**F**) in test phytoextracts treated AWFs and CWFs. Note: Statistical significance (P) calculated by one-way ANOVA followed by Dunnett’s test (for **A**, **B**, **C** and **D**) and student’s paired t test (for **E** and **F**)—*P* < 0.05 was considered as statistically significant

**Fig. 5:**
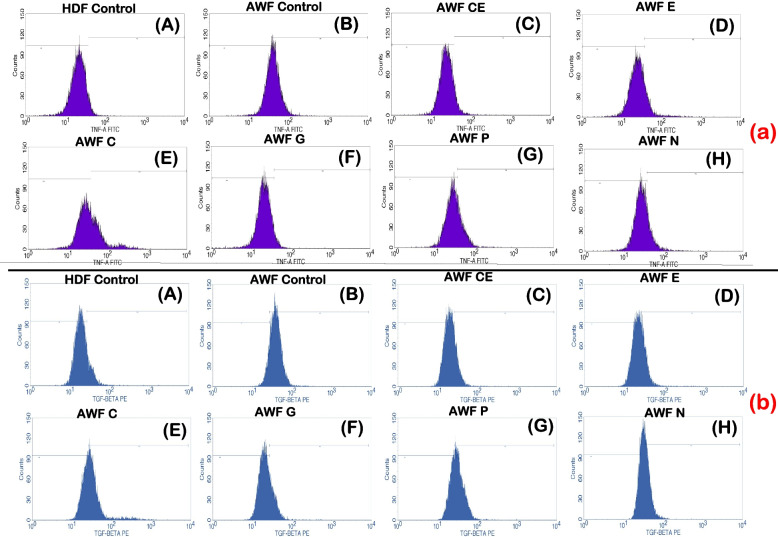
Overlaid histograms depicting (**A**) TGF-β expression observed in AWFs (**B**) TNF-α expression of AWFs (**C**) TGF-β expression observed in CWFs (**D**) TNF-α expression of CWFs in treated and untreated conditions, respectively

The anti-inflammatory activity of the test phytoextracts catechin, epicatechin, curcumin, garlic, pomegranate and neem on AWFs and CWFs were studied using flow cytometry. The pro-inflammatory cytokines TGF-β and TNF-α were used as markers and their expression levels were evaluated before and after treatment with the phytoextracts of 100 μg/ml concentration. The levels of expression of these cytokines in AWFs and CWFs were compared with that of normal HDFs post treatment with the phytoextracts. It was observed that, TGF-β expression was higher in untreated AWFs and TNF-α expression was higher in untreated CWFs, respectively (Fig. 4E and F). In addition, the overall expression of TGF-β and TNF-α was significantly (*P* < 0.05) reduced in both AWFs and CWFs after treatment with the phytoextracts. The overlaid histograms depicting TGF-β and TNF-α expression observed in AWFs and CWFs in both treated and untreated conditions is shown in Fig. 5.

### Anti-inflammatory activity in AWFs

TGF-β expression was higher in AWFs as compared to CWFs before and post treatment with phytoextracts. In AWFs, all the selected phytoextracts (catechin, epicatechin, curcumin, garlic, pomegranate and neem) showed anti-inflammatory activity by reducing the TGF-β and TNF-α expression post treatment. Percentage AWFs expressing TGF-β and TNF-α intensity is mentioned in Table 4. Interestingly, TGF-β expression was highest in pomegranate peel extract and least in catechin extract treated cells (Fig. 4A and B). Likewise, TNF-α expression was highest in curcumin extract and least in garlic extract treated cells. Therefore, of the six phytoextracts, catechin extract and garlic extract showed highest anti-inflammatory activity (*P* < 0.05) for TGF-β and TNF-α, respectively. In contrast, pomegranate peel extract and curcumin extract showed least anti-inflammatory activity (*P* < 0.05) for TGF-β and TNF-α, respectively. Finally, the anti-inflammatory effects of the test phytoextracts were compared with the untreated AWFs and untreated normal HDFs.

**Table 4:** Percentage AWFs and CWFs expressing TGF-β and TNF-α intensity

Cells	AWFs		CWFs	
Culture condition	**% Cells expressed** **TGF- β intensity**	**% Cells expressed** **TNF-α intensity**	**% Cells expressed** **TGF- β intensity**	**% Cells expressed** **TNF-α intensity**
**Untreated**	96.6 ± 0.77***	60.13 ± 2.06***	86.51 ± 0.72***	86.66 ± 1.16***
**CE**	13.3 ± 3.09***	12.46 ± 0.46***	33.1 ± 5.64***	39.01 ± 3.19***
**E**	17.6 ± 1.14***	12.87 ± 2.77***	31.42 ± 1.84***	33.53 ± 2.45***
**C**	24.3 ± 2.41***	33.38 ± 3.74***	42.42 ± 8.04***	44.06 ± 2.77***
**G**	20.8 ± 1.86***	8.183 ± 0.95***	33.88 ± 3.40***	28.05 ± 4.05***
**P**	28.1 ± 3.14***	24.25 ± 3.44***	54.08 ± 4.98***	43.87 ± 2.31***
**N**	22.3 ± 2.19***	27.28 ± 2.98***	82.14 ± 0.98	67.18 ± 2.04***

Values are expressed as the mean ± SEM (*n* = 3). Statistical significance (P) calculated by one-way ANOVA followed by Dunnett’s test—***P < 0.001 was considered as statistically significant by comparing treated group with untreated group

**Fig. 6:**
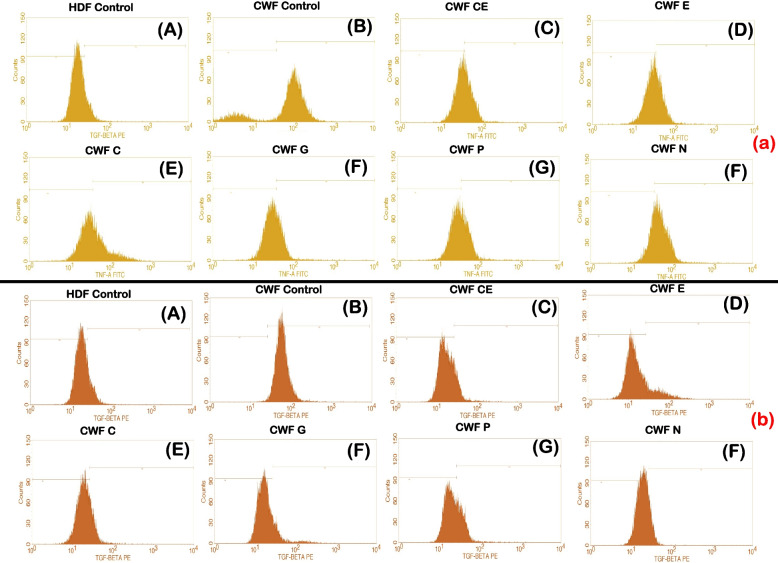
Comparative expression of TGF-β in best phytoextracts treated AWFs and CWFs v/s HDF cell line (**A**) Comparative expression of TNF-α in best phytoextracts treated AWFs and CWFs v/s HDF cell line (**B**). Note: Statistical significance (P) calculated by one-way ANOVA followed by Dunnett’s test—*P* < 0.05 was considered as statistically significant

**Fig. 7:**
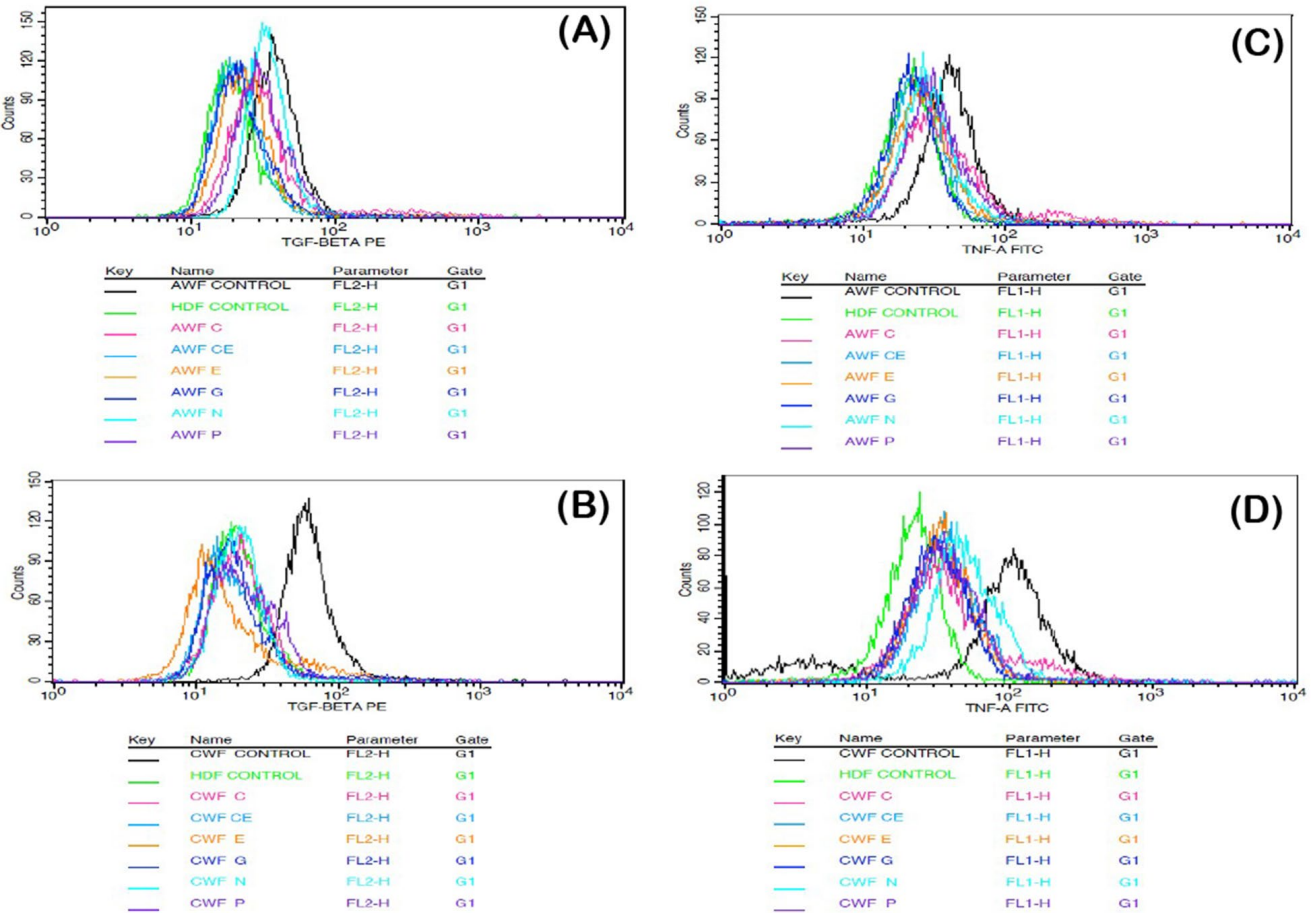
TGF-β expression histogram of test phytoextracts against AWFs (**a**) TNF-α expression histogram of test phytoextracts against AWFs (**b**) Note: A- HDF control, B- AWF Untreated, C- Catechin extract, D- Epicatechin extract, E- Curcumin extract, F- Garlic extract, G- Pomegranate extract, H- Neem Extract. TGF-β histogram of the gated AWF singlets distinguishes cells at the M1 and M2 phases. (Here M1 refers to negative expression/region and M2 refers to the Positive expression/region). Gating of M1 and M2 phases is approximate and can be refined using software (Cell Quest Pro Software, Version 6.0) analysis. % of cells observed in M2 region is considered as TGF- β expression

After catechin extract treatment, the TGF-β expression reached to almost normal HDFs levels (Fig. 6A), and after treatment with garlic extract the TNF-α expression was found to be lesser than normal HDFs (Fig. 6B). The TGF-β and TNF-α expression histograms of test phytoextracts is shown in Fig. 7A and B, respectively.

### Anti-inflammatory activity in CWFs

**Fig. 8:**
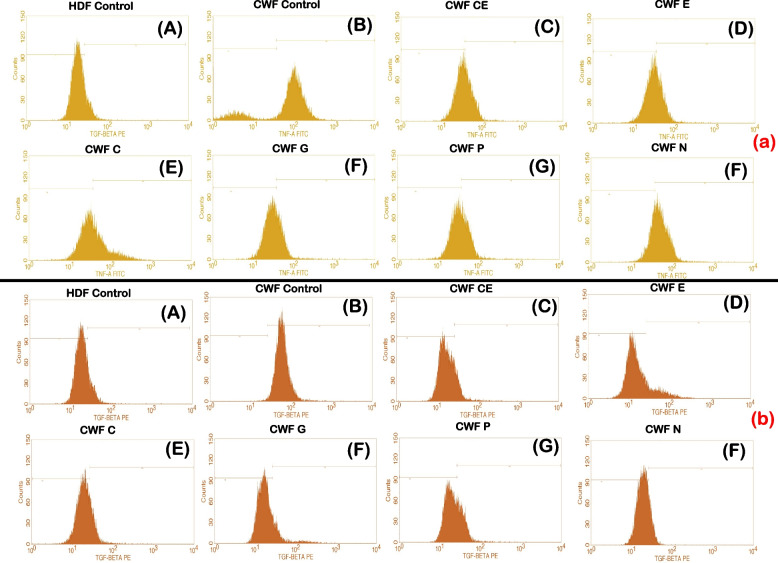
TGF-β expression histogram of test phytoextracts against CWFs (**a**) TNF-α expression histogram of test phytoextracts against CWFs (**b**). Note: A- HDF control, B- CWF Untreated, C- Catechin extract, D- Epicatechin extract, E- Curcumin extract, F- Garlic extract, G- Pomegranate extract, H- Neem Extract. TGF-β histogram of the gated CWF singlets distinguishes cells at the M1 and M2 phases. (Here M1 refers to negative expression/region and M2 refers to the Positive expression/region). Gating of M1 and M2 phases is approximate and can be refined using software (Cell Quest Pro Software, Version 6.0) analysis. % of cells observed in M2 region is considered as TGF- β expression

All the selected phytoextracts exhibited anti-inflammatory activity in CWFs against TGF-β and TNF-α. Percentage CWFs expressing TGF-β and TNF-α intensity is mentioned in Table 4. In case of CWFs, TGF-β expression was highest in neem extract least in epicatechin extract treated cells. Similarly, TNF-α expression was highest in neem extract and least in garlic extract treated cells (Fig. 4C and D). Moreover, the test phytoextracts showed higher expression of TNF-α in CWFs compared to AWFs. Therefore, of the six phytoextracts, epicatechin extract and garlic extract showed highest anti-inflammatory activity (*P* < 0.05) for TGF-β and TNF-α, respectively. However, neem extract showed least anti-inflammatory activity (*P* < 0.05) for both TGF-β and TNF-α. After treatment of CWFs with catechin, epicatechin and garlic extracts, the TGF-β and TNF-α expression was significantly (*P* < 0.05) reduced compared to untreated CWFs and was found to be lesser than untreated AWFs (Fig. 6A and B, respectively). The TGF-β and TNF-α expression histograms of test phytoextracts is shown in Fig. 8A and B, respectively.

## Discussion

The immunofluorescence staining with vimentin and α-SMA on the isolated cells (from wound tissues) revealed the presence of fibroblast cells. Vimentin is an important intermediate filament protein that forms the main structural component of mesenchymal cells especially, fibroblasts [31]. Thus, it is an extensively used morphological marker to identify fibroblasts [32]. Fibroblasts differentiates into myofibroblast like cells that morphologically resemble fibroblasts and also stain positive for α-SMA [33]. The expression of α-SMA indicate the phenotype of myofibroblasts that are predominant during wound repair and pathological conditions such as fibrosis [34]. α-SMA is used as a marker for identification of fibroblasts and myofibroblasts in many recent studies [30, 35]. Thus, in our current study, vimentin and α-SMA were used as markers to identify the isolated cells from wound tissues as fibroblasts along with morphological and spatial identification.

Toxicity of the compounds can limit itself from entering the clinical trials. Thus, it is very important for the compounds to be non-toxic to both target cell (in our case wound fibroblasts) and normal cells which is often challenging in case of chemical drugs. From the results obtained in our current study, it was observed that neem extract showed highest toxicity at a concentration of 400 μg/ml in normal HDFs. In one of the studies conducted by Jerobin et al., showed that, neem extract formulation showed significant cytotoxicity to human lymphocytes at minimum 1.2–2 mg/ml concentration and above [36]. In contrast, it was also seen that neem was considered to be safe and was used in treating diabetic foot ulcer patients by irrigating with neem leaves extract [27]. There are few studies available in literature about mammalian toxicity of neem which showed that extracts or subproducts are either less toxic or nontoxic when administered orally. The experiments on rats showed acute/mild toxicity when intramuscular injection was administered or treated by intraperitoneal route. However, higher concentrations were more toxic irrespective of the route of administration [35]. Neem, pomegranate, curcumin and epicatechin showed cytotoxicity (cell viability less than 85%) at concentrations 200 μg/ml and above with neem showing highest cytotoxicity. However, catechin and garlic extract showed cytotoxicity at 400 μg/ml concentrations. These results show that, all phytoextracts were considered to be safe for use at concentrations below 200 μg/ml based on both cell viability and IC_50_ values.

**Fig. 9:**
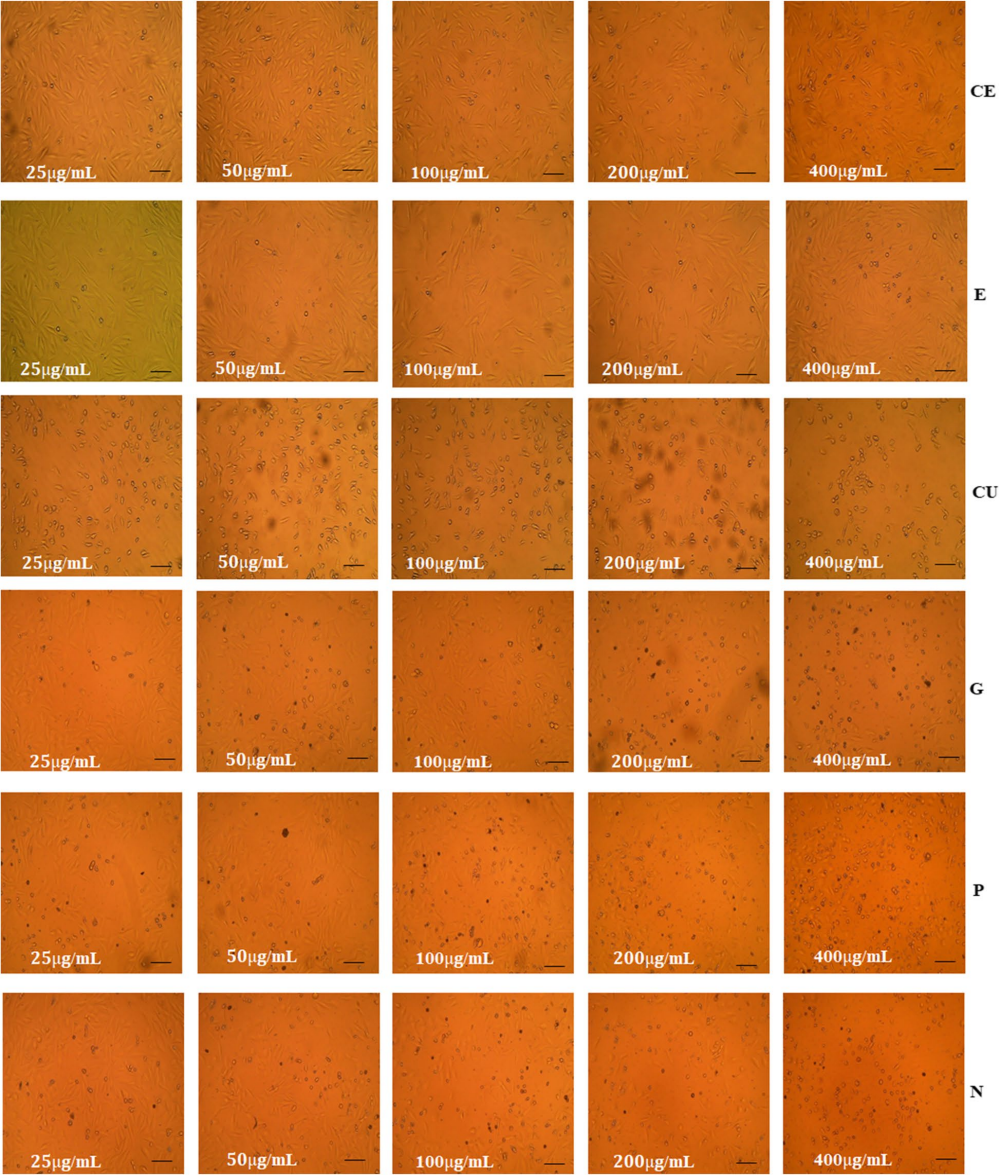
Changes in cell morphology of HDF cell line treated with various concentrations of test phytoextracts. Scale bar-100 μm

Changes in cell morphology of HDFs was observed after treatment with the phytoextracts (Fig. 9). Cell blebbing and signs of apoptosis was seen at higher concentrations resulting in loss of cell viability and ultimately cell death. Apoptosis is often represented by definite change in morphologic features such as chromatin condensation, cytoplasmic shrinkage, membrane blebbing, Endo nucleolytic degradation of genomic DNA, and the development of apoptotic bodies [37]. The observed difference in the cytotoxic effect of test phytoextracts can be attributed to the altered morphology of HDFs.

In case of AWFs, anti-inflammatory activity study showed that, of the six test phytoextracts, catechin, epicatechin and garlic extracts showed best anti-inflammatory activities against both TGF-β and TNF-α treated AWFs. A study conducted by Minhal et al. showed that topical application of 30% garlic ointment showed the presence of more proliferating fibroblasts in the scars compared to scars applied with Vaseline and increased the wound healing activity [38]. Another study showed that, garlic extracts successfully reduced the expression levels of TNF-α and significantly improved the wound closure which demonstrated its potential anti-inflammatory activity [39]. However, most studies were conducted in rats, and thus our results demonstrate their potential anti-inflammatory activity in wound associated fibroblasts. In our study, pomegranate peel and curcumin extract showed least anti-inflammatory activity against TGF-β and TNF-α treated AWFs, respectively. Few studies conducted in experimental rats showed enhanced wound healing activity using pomegranate peel (compared to pulp) mainly due to its active phytoextracts ellagic acid and other polyphenols [40, 41]. Similar results were observed while studying the ability of curcumin [42]. However, these studies were carried out in mice. Anti-inflammatory activity is exhibited by phytoextracts mostly by regulating the biochemical pathways. Usually, by decreasing the secretion of interleukin-1 (IL-1), IL-6, nitric oxide and TNF-α from the macrophages [43].

In case of CWFs, of the six test phytoextracts, catechin, epicatechin and garlic extracts showed best anti-inflammatory activities against both TGF-β and TNF-α treated CWFs. Due to its potential anti-inflammatory, antimicrobial and wound healing potential, neem leaves extract is used for treatment in diabetic foot ulcer patients [27]. However, few studies that demonstrated the potential anti-inflammatory activity of neem was carried out in rat models [44]. A recent study also observed that, catechin, epicatechin, and rutin showed safety and high efficiency in reducing hyperglycaemia by altering multiple biochemical pathways involved in hyperglycaemia [16]. Most of the studies carried out earlier were conducted in animal models.

**Fig. 10:**
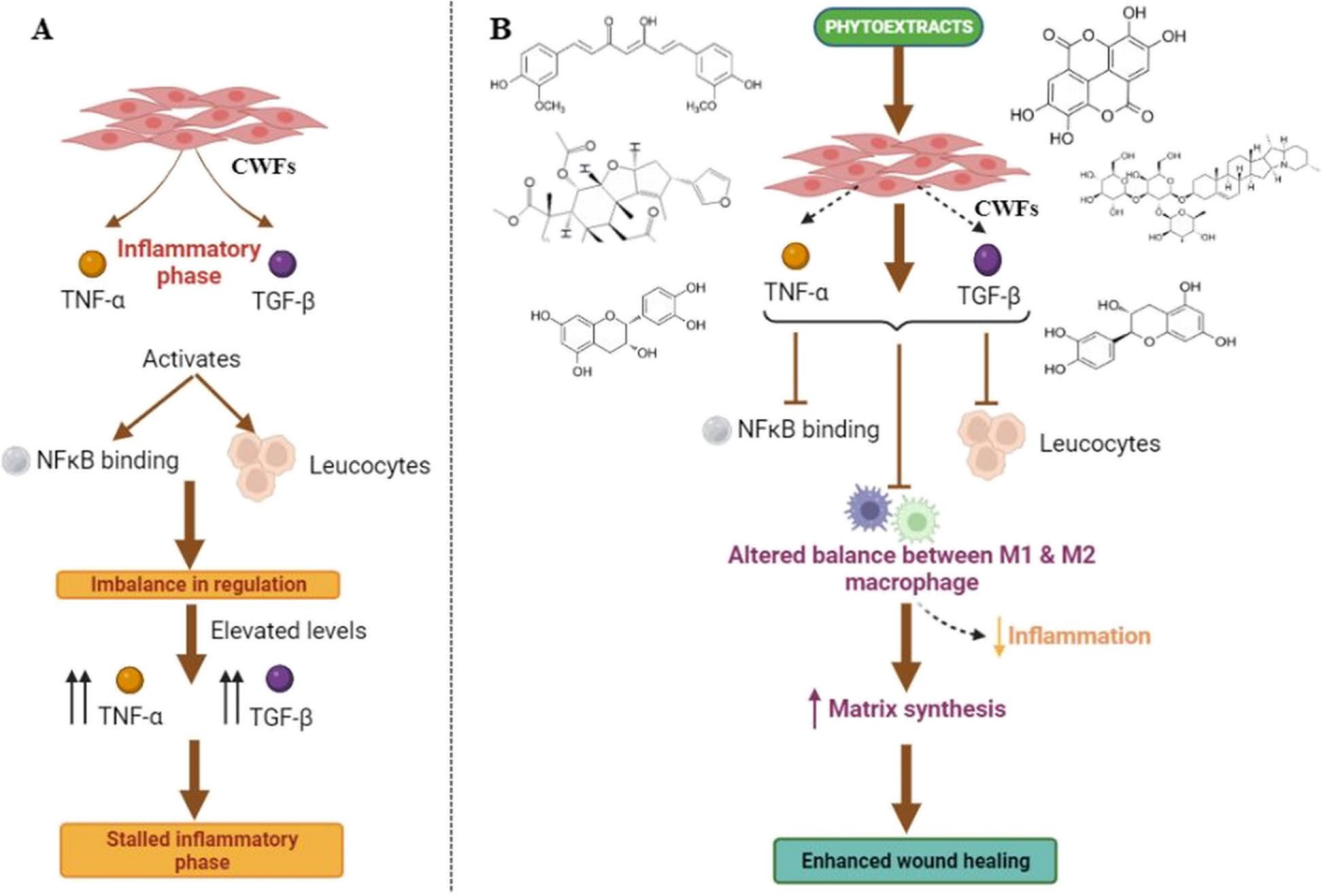
Proposed mechanism of action of anti-inflammatory activity of phytoextracts against TNF-α and TGF-β. **A** TNF-α and TGF-β, major pro-inflammatory cytokines are secreted by CWFs during inflammatory phase of wound healing. They activate NFκB binding and recruitment of leucocytes which cause imbalance in regulation of TNF-α and TGF-β levels and activity [45]. The elevated levels of TNF-α and TGF-β results in stalled inflammatory phase and impairs wound healing. **B** Treatment of chronic wounds with phytoextracts containing various key phytoconstituents synergically blunts recruitment of leucocytes and NFκB activation. This results in altered balance between M1 and M2 macrophages that reduces inflammation and regulates inflammatory phase of wound healing process. The wound healing is promoted by suppression of inflammatory parameters and increased matrix synthesis. Note: The selected phytoextracts are catechin, epicatechin, curcumin, garlic, pomegranate peel and neem. The major phytoconstituents present in these extracts are catechin, epicatechin, curcumin, allicin, ellagic acid and nimbin, respectively

The results generated in our current study are based on observations in human primary acute and chronic wound derived fibroblasts and thus may reflect on the effects of the test phytoextracts on the diseased patients as such. The mechanism of action of anti-inflammatory activity of phytoextracts against TNF-α and TGF-β is shown in Fig. 10. Thus, the selected phytoextracts in our current study proved to serve as potential drug candidates for effective acute and chronic wound healing, as they were successfully able to reduce the inflammation in both AWFs and CWFs to normal levels. However, the major phytoextracts (namely, catechin, epicatechin and garlic) that showed excellent anti-inflammatory activity in the respective cells may be considered as potential therapeutics to treat patients with acute wounds and chronic wounds safely, specifically and efficiently. However, more clinical studies in future are required to validate the results.

## Conclusion

The popular wound healing phytoextracts selected in the current study namely, catechin, epicatechin, curcumin, garlic, pomegranate peel and neem exhibited no cytotoxicity on normal HDFs. Of the six selected phytoextracts considered for protein expression studies, garlic extract showed highest cell viability followed by catechin, epicatechin, curcumin, pomegranate peel and neem extract based on its IC_50_ value. Anti-inflammatory activity of these phytoextracts in AWFs and CWFs showed that, garlic, catechin and epicatechin extracts showed excellent anti-inflammatory activities for both the cytokines TGF-β and TNF-α. After treatment of CWFs with catechin, epicatechin and garlic extracts, the TGF-β and TNF-α expression was significantly reduced compared to untreated AWFs and untreated CWFs and was close to HDF expression levels.

## Data Availability

All data generated or analysed during the current study are part of a PhD program and also, we are planning to file a patent shortly after further studies. Due to this reason, at this point we defer to deposit the information in the public repository. The data of this study shall be shared upon requesting the corresponding author.

## References

[1] Ruedrich ED, Henzel MK, Hausman BS, Bogie KM (2013). Reference gene identification for reverse transcription-quantitative polymerase chain reaction analysis in an ischemic wound-healing model. J Biomol Tech.

[2] Allman RM (1998). The impact of pressure ulcers on health care costs and mortality. Adv Skin Wound Care.

[3] Monika P, Waiker PV, Chandraprabha MN, Rangarajan A, Murthy KNC (2021). Myofibroblast progeny in wound biology and wound healing studies. Wound Repair Regen.

[4] Chen L, Cheng L, Gao W, Chen D, Wang C, Ran X (2020). Telemedicine in chronic wound management: systematic review and meta-analysis. JMIR Mhealth Uhealth.

[5] Strausberg J, Lehmann N, Kröger K, Maier I, Schneider HNW (2007). Changes in secondary care may explain increasing pressure ulcer rates in an University Clinic in Germany. Wound Manag.

[6] Diegelmann R (2004). Wound healing: an overview of acute, fibrotic and delayed healing. Front Biosci.

[7] Shukla VK, Ansari MAGS (2005). Wound Healing Research: A Perspective From India. Int J Low Extrem Wounds.

[8] Swezey L (2019). The difference between acute and chronic wounds.

[9] Falanga V (2005). Wound healing and its impairment in the diabetic foot. Lancet.

[10] Brem H, Sheehan P, Boulton AJM (2004). Protocol for treatment of diabetic foot ulcers. Am J Surg.

[11] Menke NB, Ward KR, Witten TM, Bonchev DG, Diegelmann RF (2007). Impaired wound healing. Clin Dermatol.

[12] Monika P, Chandraprabha MN (2020). Phytonanotechnology for enhanced wound healing activity. In Functional Bionanomaterials.

[13] Monika P, Chandraprabha MN, Rangarajan A, Waiker PV, Chidambara Murthy KN (2022). Challenges in healing wound: role of complementary and alternative medicine. Front Nutr.

[14] Aggarwal BB, Sundaram C, Malani N, Ichikawa H (2007). Curcumin: The Indian solid gold.

[15] Chaniad P, Tewtrakul S, Sudsai T, Langyanai S, Kaewdana K (2020). Anti-inflammatory, wound healing and antioxidant potential of compounds from Dioscorea bulbifera L. bulbils. PLoS One..

[16] Mechchate H, Es-safi I, Haddad H, Bekkari H, Grafov A, Bousta D (2021). Combination of Catechin, Epicatechin, and Rutin: Optimization of a novel complete antidiabetic formulation using a mixture design approach. J Nutr Biochem.

[17] Bae J, Kim N, Shin Y, Kim S-Y, Kim Y-J (2020). Activity of catechins and their applications. Biomed Dermatol.

[18] Prakash M, Basavaraj BV, Chidambara Murthy KN (2019). Biological functions of epicatechin: Plant cell to human cell health. J Funct Foods.

[19] Mohammadi A, Mashayekhi K, Navashenaq JG, Haftcheshmeh SM (2022). Curcumin as a natural modulator of B lymphocytes: evidence from in vitro and in vivo studies. Mini Rev Med Chem..

[20] Saifi B, Haftcheshmeh SM, Feligioni M, Izadpanah E, Rahimi K, Hassanzadeh K (2022). An overview of the therapeutic effects of curcumin in reproductive disorders with a focus on the antiinflammatory and immunomodulatory activities. Phyther Res.

[21] Mohammadian Haftcheshmeh S, Khosrojerdi A, Aliabadi A, Lotfi S, Mohammadi A, Momtazi-Borojeni AA, Pedersen SHF (2020). Immunomodulatory effects of curcumin in rheumatoid arthritis: evidence from molecular mechanisms to clinical outcomes. Reviews of physiology, biochemistry and pharmacology, vol 179.

[22] Jayaprakasha GK, Murthy KNC, Patil BS (2016). Enhanced colon cancer chemoprevention of curcumin by nanoencapsulation with whey protein. Eur J Pharmacol.

[23] Akbik D, Ghadiri M, Chrzanowski W, Rohanizadeh R (2014). Curcumin as a wound healing agent. Life Sci.

[24] Alhashim M, Lombardo J (2020). Effect of Topical Garlic onWound Healing and Scarring: A Clinical Trial. Dermatologic Surg.

[25] Mahdi Mirghazanfari S, Nassireslami E, Sheikh Asadi M, Dadpay M (2018). Evaluation of wound healing activities of pomegranate (Punica granatum-Lythraceae) peel and pulp. J Res Med Dent Sci.

[26] Chundran N, Husen I, Journal IR-AM, 2015 U (2015). Effect of Neem leaves extract (Azadirachta Indica) on wound healing. Althea Med J..

[27] Srinivasan JM (2020). Effect of neem leaves extract irrigation on the wound healing outcome in nurse managed diabetic foot ulcers. Natl J Physiol Pharm Pharmacol.

[28] Barrientos S, Stojadinovic O, Golinko MS, Brem H, Tomic-Canic M (2008). Growth factors and cytokines in wound healing. Wound Repair Regen.

[29] Gooyit M, Peng Z, Wolter WR, Pi H, Ding D, Hesek D (2014). A chemical biological strategy to facilitate diabetic wound healing. ACS Chem Biol.

[30] Monika P, Chandraprabha MN, Chidambara Murthy KN, Annapoorni Rangarajan P. Veena Waiker, Sathish M. Human primary chronic wound derived fibroblasts demonstrate differential pattern in expression of fibroblast specific markers, cell cycle arrest and reduced proliferation. Exp Mol Pathol. 2022;127:104803. 10.1016/j.yexmp.2022.104803 35679887

[31] Franke WW, Hergt M, Grund C (1987). Rearrangement of the vimentin cytoskeleton during adipose conversion: Formation of an intermediate filament cage around lipid globules. Cell.

[32] Ostrowska-Podhorodecka Z, McCulloch CA (2021). Vimentin regulates the assembly and function of matrix adhesions. Wound Repair Regen.

[33] Oda D, Gown AM, Vande Berg JS, Stern R (1988). The fibroblast-like nature of myofibroblast. Exp Mol Pathol.

[34] Zhang HY, Gharaee-Kermani M, Zhang K, Karmiol S, Phan SH (1996). Lung fibroblast α-smooth muscle actin expression and contractile phenotype in bleomycin-induced pulmonary fibrosis. Am J Pathol.

[35] Braga TM, Rocha L, Chung TY, Oliveira RF, Pinho C, Oliveira AI (2021). Azadirachta indica A. Juss. In Vivo Toxicity. Molecules.

[36] Jerobin J, Makwana P, Suresh Kumar RS, Sundaramoorthy R, Mukherjee A, Chandrasekaran N (2015). Antibacterial activity of neem nanoemulsion and its toxicity assessment on human lymphocytes in vitro. Int J Nanomedicine.

[37] Rogers CS, Yedjou CG, Sutton DJ, Tchounwou PB (2014). Vitamin D3 potentiates the antitumorigenic effects of arsenic trioxide in human leukemia (HL-60) cells. Exp Hematol Oncol.

[38] Alhashim M, Lombardo J (2018). Mechanism of Action of Topical Garlic on Wound Healing. Dermatologic Surg.

[39] Zaenal, Mustamin R, Taher R, Mallongi A (2019). Efficacy of topical cream of garlic extract (Allium Sativum) on Wound Healing in Experimental Mice using Aa Acute Wound Modeling: Determination of Expresión of Tumor Necrotic Factor (TNF-α). Indian J Public Heal Res Dev..

[40] Lukiswanto BS, Miranti A, Sudjarwo SA, Primarizky H, Yuniarti WM (2019). Evaluation of wound healing potential of pomegranate (Punica granatum) whole fruit extract on skin burn wound in rats (Rattus norvegicus). J Adv Vet Anim Res.

[41] Mahdi Mirghazanfari S, Nassireslami E, Sheikh Asadi M, Dadpay M (2018). Evaluation of wound healing activities of pomegranate (Punica granatum-Lythraceae) peel and pulp. J Res Med Dent Sci.

[42] Yen YH, Pu CM, Liu CW, Chen YC, Chen YC, Liang CJ (2018). Curcumin accelerates cutaneous wound healing via multiple biological actions: The involvement of TNF-α, MMP-9, α-SMA, and collagen. Int Wound J.

[43] Nazir Y, Linsaenkart P, Khantham C, Chaitep T, Jantrawut P, Chittasupho C (2021). High Efficiency In Vitro Wound Healing of Dictyophora indusiata Extracts via Anti-Inflammatory and Collagen Stimulating (MMP-2 Inhibition) Mechanisms. J Fungi.

[44] Banerjee K, Chatterjee M, Sandur VR, Nachimuthu R, Madhyastha H, Thiagarajan P (2021). Azadirachta indica A. Juss (Neem) oil topical formulation with liquid crystals ensconcing depot water for anti-inflammatory, wound healing and anti-methicillin resistant Staphylococcus aureus activities. J Drug Deliv Sci Technol..

[45] Wise SC, Burnett J, Coupar J, Yang X, Chen Z, Van Waes C (2013). TGF-β and NF-κB signal pathway cross-talk is mediated through TAK1 and SMAD7 in a subset of head and neck cancers. Oncogene..

